# Activin B and Activin C Have Opposing Effects on Prostate Cancer Progression and Cell Growth

**DOI:** 10.3390/cancers15010147

**Published:** 2022-12-27

**Authors:** Karen L. Reader, Simon John-McHaffie, Sylvia Zellhuber-McMillan, Tim Jowett, David G. Mottershead, Heather E. Cunliffe, Elspeth J. Gold

**Affiliations:** 1 Department of Pathology, University of Otago, Dunedin 9054, New Zealand; 2 Department of Mathematics and Statistics, University of Otago, Dunedin 9054, New Zealand; 3 School of Pharmacy and Bioengineering, Keele University, Newcastle-under-Lyme ST5 5BG, UK; 4 Department of Anatomy, University of Otago, Dunedin 9054, New Zealand

**Keywords:** prostate cancer, biomarker, Gleason grade, transforming growth factor-β family, activin, INHBA, INHBB, INHBC

## Abstract

**Simple Summary:**

Prostate cancer is one of the leading causes of death in men. Current methods for grading and determining treatment options are not as accurate as they need to be with some men either undergoing unnecessary, life-changing treatment or not being treated and developing late-stage disease. We assessed the effect of proteins called activins on the growth of prostate cell lines and examined their expression in prostate cancer biopsy samples from patients with different grades of tumor. Activin B and activin C were shown to have increased and decreased expression, respectively, in higher grade prostate cancer and to have opposing effects on prostate cell growth and migration. Therefore, these proteins are potential markers for distinguishing aggressive from indolent prostate cancer and may also provide novel targets for prostate cancer treatment.

**Abstract:**

Current prognostic and diagnostic tests for prostate cancer are not able to accurately distinguish between aggressive and latent cancer. Members of the transforming growth factor-β (TGFB) family are known to be important in regulating prostate cell growth and some have been shown to be dysregulated in prostate cancer. Therefore, the aims of this study were to examine expression of TGFB family members in primary prostate tumour tissue and the phenotypic effect of activins on prostate cell growth. Tissue cores of prostate adenocarcinoma and normal prostate were immuno-stained and protein expression was compared between samples with different Gleason grades. The effect of exogenous treatment with, or overexpression of, activins on prostate cell line growth and migration was examined. Activin B expression was increased in cores containing higher Gleason patterns and overexpression of activin B inhibited growth of PNT1A cells but increased growth and migration of the metastatic PC3 cells compared to empty vector controls. In contrast, activin C expression decreased in higher Gleason grades and overexpression increased growth of PNT1A cells and decreased growth of PC3 cells. In conclusion, increased activin B and decreased activin C expression is associated with increasing prostate tumor grade and therefore have potential as prognostic markers of aggressive prostate cancer.

## 1. Introduction

Prostate cancer (PCa) is the second most commonly diagnosed male cancer and the fifth leading cause of cancer deaths in men [1]. Gleason scoring of prostate needle biopsies is currently the best method for determining tumor aggressiveness and metastatic potential, however it is unable to provide a definitive signal about an individual patient’s prognosis. Two studies have reported that a significant proportion of patients meeting the criteria for active surveillance who chose to have a radical prostatectomy, had more advanced cancer than predicted by the Gleason score [2,3]. Prostate tumors are heterogeneous, and biopsies may omit to sample regions containing higher-grade tumour, leading to their misclassification as low-risk cancer. The morphology of the biopsy provides a snapshot of the tumor in time but cannot confirm if a low-grade tumor will remain indolent or grow rapidly. For example, Gleason pattern 3 tumors have been shown to be of the same clonal origin to adjacent Gleason pattern 4 tumors and are likely to develop into higher grade cancers [4]. Changes to protein expression can occur throughout the cancer, irrespective of the morphology or grade of the tumor, and can potentially provide information about the overall tumor biology and thus better predict the rate of tumor growth. Therefore, the identification of biomarkers that can be easily quantified in biopsy cores without the need to differentiate between different cell types and tumor grades, that can predict clinical outcome, would aid clinical assessment of progression risk. However, we have a limited understanding of the molecular changes occurring in PCa and which genes are important for driving cancer progression [5]. Recent studies have attempted to identify biomarkers that can predict PCa outcome and while some clinical tests have been developed these are yet to be proven to change clinical decision making and patient outcome [6,7]. Despite numerous large-scale genomic studies, only a small number of single nucleotide polymorphisms have been associated with clinical outcome in PCa [5]. 

Members of the transforming growth factor-β (TGFB) family, including ligands, receptors and signaling molecules, are known to play an important role in prostate homeostasis and are dysregulated in a number of different cancers, including PCa [8,9,10]. Activins are members of the TGFB family and consist of homo- or hetero-dimers of the inhibin β subunits (βA, βB, βC and βE) to form, for example, activin A (βA:βA), activin B (βB:βB) or activin AB (βA:βB). The subunit gene names are inhibin-βA (INHBA; activin A), INHBB (activin B), INHBC (activin C) and INHBE (activin E). Activins A and B initiate canonical signaling by binding to type 2 serine/threonine kinase receptors which then recruit type 1 receptors to the receptor complex at the cell surface. This initiates binding and phosphorylation of the transcription factors, mothers against decapentaplegic homolog 2 and 3 (SMAD2 and SMAD3) which, along with the co-factor SMAD4, translocate to the nucleus to initiate gene transcription [11]. Other non-canonical signaling pathways have also been shown to be involved in activin A signaling in different cell types, such as the p38 MAP kinase (MAPK) and c-Jun amino-terminal kinase (JNK) pathways. For a review of the function and roles of activins and inhibins see Namwanje, Brown [12]. 

There is some evidence that activins are involved in the regulation of PCa growth. Activin A (INHBA) has been shown to inhibit proliferation of LNCaP cells and both follistatin (FST) and activin C (INHBC) can block INHBA growth inhibition of these cells [13,14]. Chen et al. [15] have reported that high expression of INHBA is associated with reduced survival and in metastasis compared to corresponding primary sites in prostate cancer. However, little is known about the effect of INHBB or INHBC on the growth of normal and malignant prostate cells. We have recently reported that INHBC protein expression was increased in human PCa tissue from cases with extra-capsular spread when compared to normal prostate tissue or organ-confined cases [16]. These results need to be validated in a greater number of cases, and the expression of other TGFB family ligands, receptors and signaling molecules should be examined. 

The first aim of this study was to determine if the level of protein expression of activins, their receptors and signaling molecules differ between human PCa biopsy samples with different Gleason patterns using immunohistochemistry with commercially available antibodies. A second aim was to examine the effect of recombinant INHBA, INHBB and INHBC on proliferation of normal, immortalized prostate cells (PNT1A) and PCa cell lines (DU145, LNCaP and PC3), and to compare levels of activin protein expression between these different cell lines. The third aim was to examine the effect of ectopic INHBB or INHBC overexpression on growth and migration of prostate cell lines.

## 2. Materials and Methods

All reagents were supplied by Thermo Fisher Scientific, Auckland, NZ unless otherwise stated. 

### 2.1. Immunohistochemistry

Tissue microarray (TMA) slides (PR483b, PR752, PR8011a; US Biomax, Derwood, MD, USA) containing cores from human cases of primary prostate adenocarcinoma (134 cores, 88 cases) and normal prostate tissue (15 cores, 13 cases) were immuno-stained following methods described by Marino et al. [17]. The antibodies used for immunohistochemistry are listed in Table 1. Secondary antibody detection was performed using the DAKO EnVision + Dual Link System-HRP Rabbit/Mouse (DAKO, Glostrup, Denmark) with a 15 min diaminobenzidine (DAB) incubation followed by hematoxylin counterstaining. Primary antibody concentrations were optimized using sample TMA slides (US Biomax; T191) and sections of normal human prostate tissue. Negative control mouse and normal goat antibodies were used on either a TMA slide (T191), or sections of human prostate, at the highest concentration used for the corresponding species and antibody type, with either the secondary antibody used above, or polyclonal rabbit anti-goat IgG/HRP (DAKO). These slides were negative for DAB staining. Immunohistochemistry was performed for each individual antibody on all three TMA slides in a single run to allow immuno-reactive scores to be compared across TMAs. Immuno-reactive scores were semi-quantified digitally using Fiji software [18] according to the methods previously described by Helps et al. [19] and Fuhrich et al. [20]. Briefly, TMAs were imaged using an Aperio Digital Slide Scanner (Leica Biosystems, Melbourne, Australia) and individual core images thresholded (minimum) using Fiji to select only the area of the tissue. Hematoxylin and DAB staining were digitally separated using the color deconvolution plugin HDAB [21] and the DAB image thresholded (default) to select only the area stained by DAB. This was overlaid on the selected area of the tissue and the percent area of DAB stained tissue calculated by the software. Histogram analysis was carried out on the deconvoluted DAB image and the intensity of DAB staining calculated by the formula:

$$\mathrm{DABwt}\%=\left(\frac{\mathrm{DABwt}}{\Sigma \left(\mathrm{Histogram}\;\mathrm{Value}\;\mathrm{Counts}\right)\times 255}\right)\times 100$$

Immuno-reactive scores (IRS) were calculated for each TMA core by multiplying the % area of DAB staining by the DABwt%. A TMA slide stained for INHBC was also manually scored by two experienced, blinded, independent observers as described by Marino et al. [22] and the IRS compared between the two methods. Results from the manual and digital scoring methods were significantly correlated with a Pearson R^2^ of 0.7227 and *p* < 0.001.

### 2.2. Online Analysis of Activin mRNA Expression

An online analysis of publicly available PCa patient datasets was performed with cumulated data from Oncomine (Compendia Bioscience https://www.oncomine.org/resource/main.html accessed on 19 May 2021). A FireBrowse analysis of PCa patient datasets from The Cancer Genome Atlas (TCGA; data version 2016-01-28) that compared gene expression between normal and PCa samples was also performed. Presented data is modified from Oncomine or FireBrowse. The FireBrowse analysis did not provide statistical significance values. 

### 2.3. Recombinant INHBC Expression and Purification

Commercially available recombinant INHBC, consisting of the mature region only, did not alter PCa cell proliferation in our assays, whereas full-length, unpurified INHBC had been previously shown to alter the effect of INHBA on LNCaP cell proliferation [13]. Therefore, we produced full length, purified, recombinant INHBC to examine the effect of INHBC on PCa cell proliferation. An expression cassette was synthesized encoding the wild-type sequence of human *INHBC* modified to incorporate the rat serum albumin signal sequence at the N-terminal end, followed by a His8 affinity tag for purification and a Strep II tag to allow detection of the proregion (GenScript, Piscataway, NJ, USA). The cDNA fragment was cloned into the pEFIRESp expression vector [23] and the plasmid transfected into CHO-K1 cells using Lipofectamine 3000 following the manufacturer’s instructions. Stable cell lines (polyclonal) were established using puromycin selection (Sigma, Auckland, New Zealand). Cells were grown to near confluence and the growth media replaced with serum-free production media consisting of DMEM with 0.1 mg/mL BSA (Sigma), 2 mM GlutaMAX, 100 IU/mL penicillin and 100 μg/mL streptomycin, and cultured for 48 h. Production media was harvested, and INHBC protein purified using HisPur NiNTA resin according to the methods described by Mottershead et al. [24]. The presence of the imidazole elution buffer was found to increase growth of LNCaP cells and inhibit growth of the other prostate cell lines and was therefore removed by dialyzing the INHBC preparation in three changes of Dulbecco’s PBS using a Slide-A-Lyzer MINI Dialysis Device (3.5K MWCO). SDS-PAGE and Western blotting was performed on purified INHBC under reducing conditions, according to the methods described by Marino et al. [17]. Intercept blocking buffer (Millenium Science, Auckland, New Zealand) was used for the blocking steps and dilution of primary and secondary antibodies. Nitrocellulose membranes were incubated overnight with a 1:1000 dilution of INHBC antibody (Abcam ab73904; 0.5 μg/mL). Secondary antibody detection was performed using IRDye 800CW goat anti-mouse IgG1 (Li-Cor, Millennium Science, Auckland, New Zealand) and imaged with an Odyssey Classic scanner and Image Studio Lite software. The concentration of the purified INHBC mature region monomer was estimated by Western blot analysis relative to known amounts of recombinant human INHBC (R&D Systems, Minneapolis, MN, USA). 

### 2.4. Prostate Cell Lines

Four human prostate cell lines were used in these studies: PNT1A (Sigma; 95012614), LNCaP (ATCC; CRL-1740), DU145 (ATCC; HTB-81) and PC3 (ATCC; CRL-1435). PNT1A cells are immortalized prostatic epithelial cells that represent normal prostate epithelium [25]. LNCaP, DU145 and PC3 cells originate from prostate carcinoma metastases from lymph node, brain and bone, respectively. All PCa cell lines in our laboratory have been recently validated using STR profiling. All cells were maintained in DMEM with 10% fetal bovine serum (FBS) (Moregate Biotech, Hamilton, New Zealand), 2 mM GlutaMAX-1, 100 U/mL penicillin and 100 μg/mL streptomycin and incubated at 37 °C in a humidified atmosphere of 5% CO_2_ in air. FBS was heat-inactivated for 30 min at 56 °C.

### 2.5. Stable Transfection of Prostate Cell Lines with INHBB and INHBC

The human INHBB pCMV3-SP-N-FLAG expression plasmid (Cat# HG10814-NF; Sino Biological, Beijing, China), the pCMV3-SP-N-FLAG negative control vector (Cat# CV020; Sino Biological), the modified human INHBC pEFIRES expression plasmid (as above) or the pEFIRES negative control vector (Cat#1849; GenScript) were transfected into PNT1A and PC3 cells. Prior to transfection, plasmid stocks were prepared by transforming the DNAs into chemically competent DH5alpha *E. coli* prepared using the Inoue method [26], and plasmid minipreps were validated by restriction endonuclease digestion. Bulk DNA was then isolated for transfection using the Qiagen endotoxin-free Plasmid Maxi Kit (Cat#12362; Qiagen, Auckland, New Zealand). Prostate cells were seeded into 6-well plates with 300,000 PC3 cells or 600,000 PNT1A cells per well, and allowed to attach overnight. During transfection, growth media was replaced with Opti-MEM 30 min before transfection. Plasmid DNA (5 µg) mixed with Lipofectamine 3000 (3.75 µL, Invitrogen) was added and cells were incubated for 4 h at 37 °C in 5% CO_2_ in air. After incubation, the Opti-MEM and DNA/Lipofectamine mix was replaced with growth media containing 20% FBS and incubated at 37 °C, 5% CO_2_ for 48 h. After the 48 h growth period, stably transfected cells were selected for using hygromycin (pCMV3) or puromycin (pEFIRES) following optimization of the concentration for each antibiotic and cell line to give 100% cell death within 10 days.

### 2.6. Western Blot Quantitation of Activins in Prostate Cell Lines

Protein isolation was performed as described by Marino et al. [17] from up to three separate passages of each cell line (PNT1A, LNCaP, PC3, cell lines overexpressing INHBB or INHBC, and empty vector controls) and quantified using the Pierce BCA Protein Assay Kit (Life Technologies, Auckland, New Zealand). For each cell line, 60 μg of protein from each cell passage was separated on a 12% SDS-PAGE acrylamide gel, under reducing conditions, and Western blotting analysis performed as described above. This was repeated in three separate Western blots for each cell lysate sample. Proteins were detected with the primary antibodies and Li-Cor secondary antibodies listed in Table 2. Relative signal intensities were compared between cell lines for each protein, normalized to GAPDH, using the Odyssey infra-red imaging system and Image Studio Lite software (Li-Cor).

### 2.7. Cell Growth Assays

The effect of recombinant activins, signaling pathway inhibitors and overexpression of activins on the growth of human prostate cell lines was tested using an MTS colourimetric cell proliferation assay (CellTiter 96). Cells were cultured for 24 h in 96-well plates in DMEM with 5% FBS with 5000 cells per well. Media was then replaced with either DMEM with 2% FBS (control) or the same medium containing recombinant human INHBC (either our full-length INHBC or mature region from R&D Systems; 1629-AC), human INHBA (mature region, R&D systems; 338-AC), human INHBB (mature region, R&D systems; 659-AB), SB431542 ALK4/5/7 inhibitor (Tocris, In Vitro Technologies, Auckland, New Zealand), LY294002 PI3K/AKT inhibitor (Tocris), SB202190 MAPK inhibitor (Tocris), LDN193181 ALK2/3 inhibitor (Selleckchem #S2618) or DMSO vehicle control. Cells were incubated for either 72 h (LNCaP, PC3) or 144 h (PNT1A, DU145) to allow for the different growth rates of the cell lines. Treatments were replicated in three or six wells using a minimum of three different cell passages for each cell line. At the end of the culture period, 20 μL Promega CellTiter 96 AQueous One solution (In Vitro Technologies, Auckland, New Zealand) was added to each well and incubated for 1 h at 37 °C and the absorbance read at a wavelength of 490 nm in a VICTOR X4 multimode plate reader (PerkinElmer, Buckinghamshire, UK).

### 2.8. Migration Assays

To determine if overexpression of INHBB or INHBC altered cell migration, cells were seeded in 8 µm pore transwell permeable inserts (Corning, #3428) in 24-well companion plates (Falcon, 353504). Cells were seeded at either 25,000 PNT1A or 50,000 PC3 cells per insert and were cultured for 24 h in DMEM with 2% FBS. After incubation, inserts were washed with PBS and cells remaining on the top of the insert were removed. Migrated cells were fixed using 100% methanol for 25 min, stained overnight with 0.5% (*w*/*v*) crystal violet, washed and air-dried. Five separate fields of view per insert were imaged at 20× magnification using a DMi1 microscope with Leica Application Suite, version 3.2.0 (Leica Microsystems, Balgach, Switzerland). Cell counts were taken as the total of all stained cells in five fields of view, and were performed manually. Three independent experiments were performed using separate cell passages with triplicate inserts.

### 2.9. Statistical Analysis

All statistical analysis was carried out using R software [27]. Three different general methods were used. A General linear model (GLM) assuming a negative binomial response distribution was used to fit the Western blot normalised fluorescent intensity data for the different PCa cell lines. This model was able to accommodate the tendency for the variance of the fluorescence values to increase with increasing mean. Mean fluorescence was modelled using a linear function of the main effects, cell line and activin. The interaction effect between cell line and activin was excluded from the final model because it did not significantly improve the main effects only model. The model was fitted using the *glm.nb* function from the *MASS* package [28]. 

Generalised least squares (GLS) models were fitted to the immunohistochemistry IRS data and the MTS absorbance data for the modified PC3 cells treated with ALK2/3 inhibitor. The GLS method was used to account for differences in measurement variance across the different treatments. For the IRS data, both the mean and variance was modelled as a function of the “Highest Gleason” treatment effect. The models were fitted using the *gls* function from the *nlme* package [29].

The normal linear model (NLM) was used to fit models to absorbance data from the cell proliferation and migration assays. In all of these models, in addition to the treatment effect of interest, the date of testing was included as a predictor variable to control for any variance in experiment conditions or cell pools across the different dates. The models were fitted using the *lm* function [27]

A NLM with fluorescence fold-change response was found to be the most efficient way to model the Western blot data from the transfected cell lines. Individual fold-change values were calculated by dividing the INHBB or INHBC transfected cell line fluorescent intensities by the empty vector control values for each Western blot date. This approach helped to control for the variance in Western blot intensities across the different dates. The model was fitted using the *lm* function [27].

The validity of the underlying assumptions of all final models were tested using residual diagnostics and were found to be acceptable. All post hoc statistical testing (including adjustment for multiple comparisons) and means estimation was preformed using the R package *emmeans* [30].

## 3. Results

### 3.1. Immuno-Reactive Scores in Relation to Gleason Score

**Figure 1:**
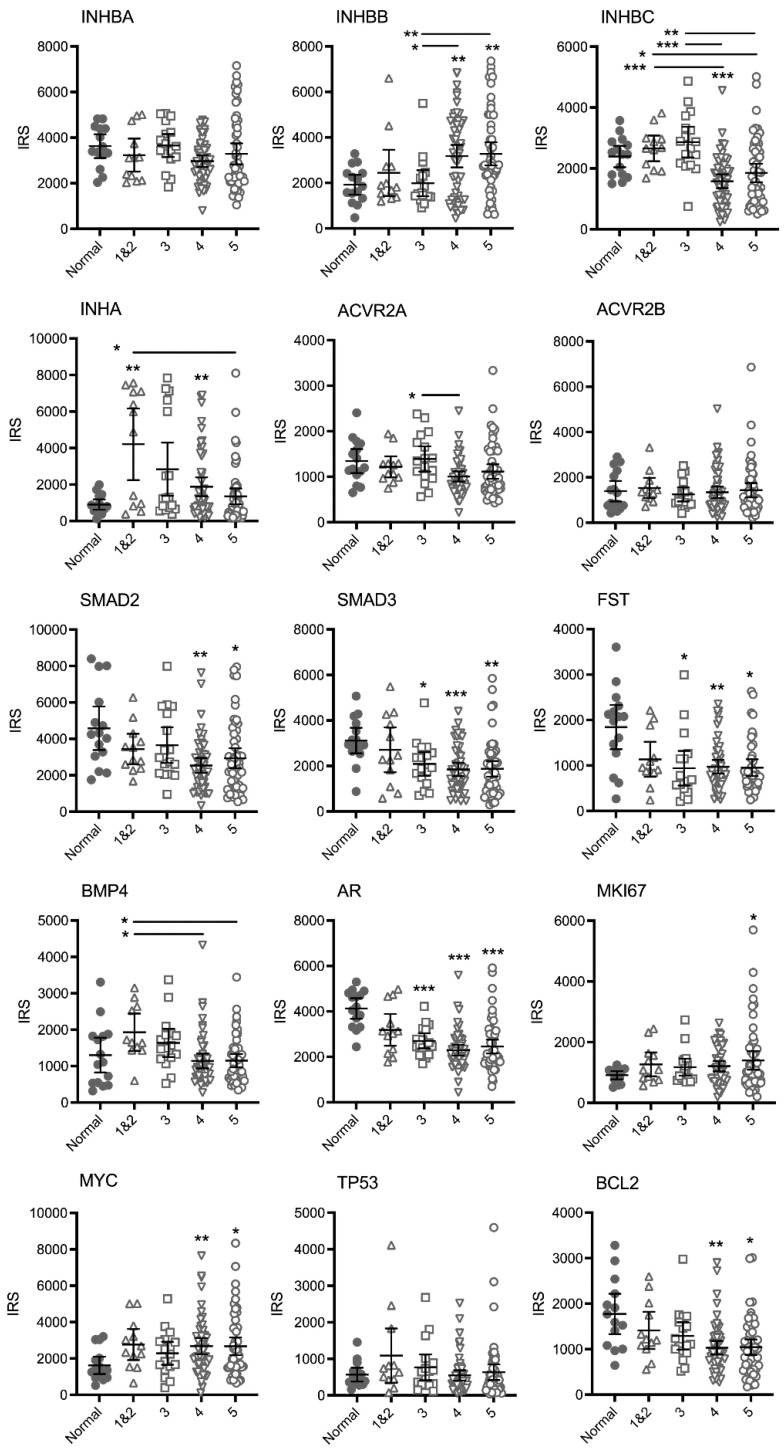
Graphs of IRS for individual TMA cores immunostained for TGFB family and other proteins relative to the highest Gleason pattern present. Bars represent mean +/− 95% confidence limits. Asterisks alone indicate significant differences compared to normal tissue and asterisks beside horizontal lines above bars indicate differences between Gleason patterns. * *p* < 0.05, ** *p* < 0.01, *** *p* < 0.001.

Gleason scores were provided for each adenocarcinoma patient core by US Biomax and the IRS were analysed relative to the highest Gleason pattern present in each core. Gleason patterns 1 and 2 were combined into one group due to the low number of cases. Graphs of each protein showing the IRS for each core and Gleason pattern are presented in Figure 1. Representative images of immunohistochemical staining for each protein in normal and high-grade PCa tissue cores are presented in Supplementary Figure S1. 

There were no differences in mean IRS for INHBA, however, expression varied greatly in the Gleason pattern 5 cases with a cluster of cores with higher IRS. For INHBB the mean IRS for pattern 4 and pattern 5 cases was greater than the normal and pattern 3 cases. The IRS in the higher-grade cores was variable and appeared to separate into clusters of either high or low IRS. In contrast, the mean IRS for INHBC Gleason pattern 4 was significantly lower than normal tissue, while both patterns 4 and 5 were lower than patterns 1&2 and pattern 3. The mean IRS for INHA (inhibin α) in Gleason pattern 1&2, 3 (*p* = 0.0504) and 4 tumors was higher than normal tissue. However, the IRS were variable and again, the cases segregated into clusters of high or low scores. There was only one significantly different group in the activin receptor 2A (ACVR2A) stained cases (pattern 4 less than pattern 3) and no differences between groups for ACVR2B indicating that receptor expression does not change with increasing Gleason pattern. Expression of SMAD2, SMAD3, follistatin (FST), androgen receptor (AR) and BCL2 decreased while MYC and marker of proliferation Ki-67 (MKI67) expression increased in the higher Gleason patterns compared to normal tissue with no differences between Gleason groups. Bone morphogenetic protein 4 (BMP4) expression appeared to increase in pattern 1&2 (not significant) compared to normal tissue and then decrease again in patterns 4 and 5. Tumour protein p53 (TP53) expression did not change with increasing Gleason pattern.

Spearman correlation coefficient analysis showed significant positive correlation in IRS between most of the proteins examined (data not shown). There was a general pattern of an increase in IRS for INHBB and a decrease in INHBC with increasing Gleason pattern and these were the only two proteins to have a negative Spearman correlation coefficient r-value (−0.253, *p* = 0.014).

### 3.2. INHBB and INHBC RNA Expression in Prostate Tissue

**Figure 2:**
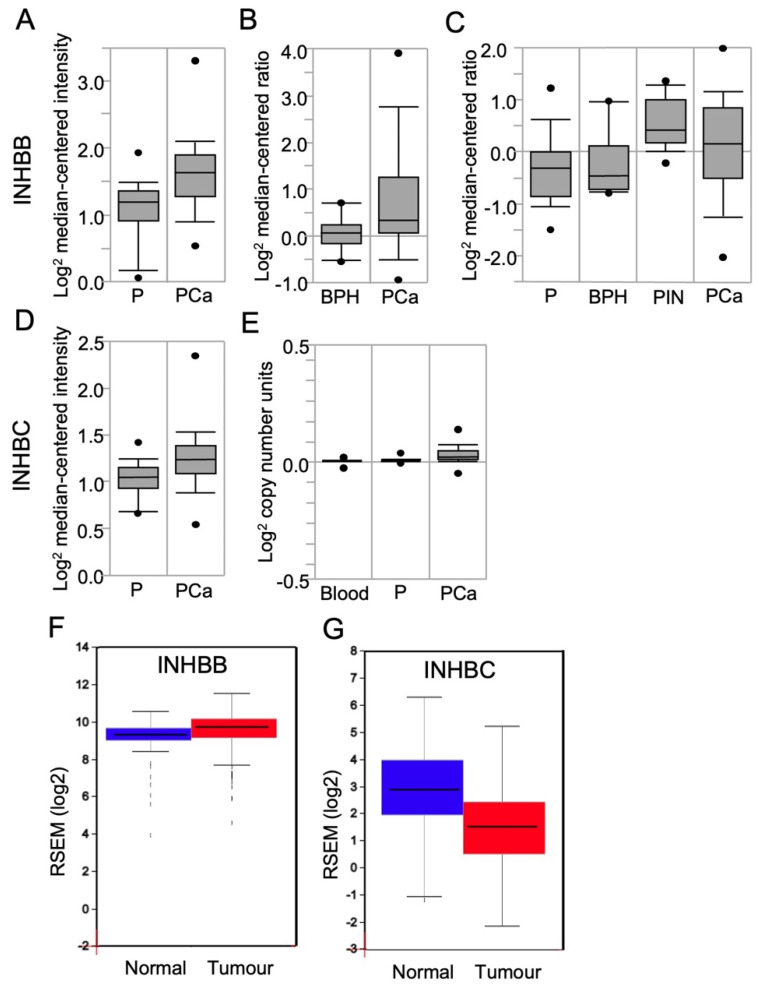
Box plots derived from gene expression data in Oncomine or FireBrowse, comparing results for human prostate expression of INHBB and INHBC. INHBB expression was shown in data sets from (**A**) Yu Prostate, comparing the prostate gland (P; *n* = 23), and prostate carcinoma (PCa; *n* = 65); (**B**) Luo Prostate, comparing benign prostatic hyperplasia (BPH; *n* = 9) and PCa (*n* = 16); (**C**) Tomlins Prostate data, comparing the normal prostate (P; *n* = 25), BPH (*n* = 10), prostatic intraepithelial neoplasia (PIN; *n* = 13), and PCa (*n* = 46). INHBC expression was shown in data sets from (**D**) Liu Prostate data, comparing prostate gland samples (P; *n* = 13) and PCa (*n* = 44); (**E**) The Cancer Genome Atlas (TCGA) comparing blood (*n* = 148), the prostate gland (P; *n* = 61) and PCa (*n* = 45). (**F**) FireBrowse RNASeq data comparing INHBB expression in normal prostate samples (blue; *n* = 52) and tumour samples (red; *n* = 498). (**G**) FireBrowse RNASeq data comparing INHBC expression in normal prostate samples (blue; *n* = 45) and tumour samples (red; *n* = 457). Figures are presented as box plots. The box midline is the median, top and bottom of the box represent 75th and 25th percentiles, and top and bottom whiskers represent 90th and 10th percentiles.

To determine if the differences in INHBB and INHBC protein expression observed between tumours with different Gleason patterns corresponded to differences in RNA expression in PCa, the Oncomine cancer microarray database was explored. Three studies were identified that compared INHBB gene expression in normal and pathological prostate tissue cases [31,32,33]. The Yu dataset showed a 1.59-fold increase (*p* < 0.0001) in INHBB expression in prostate carcinoma compared to healthy prostate (Figure 2A). The Luo dataset (Figure 2B) also showed increased INHBB expression (fold change = 1.630) in prostate carcinoma when compared to benign prostatic hyperplasia (BPH; *p* = 0.024). The Tomlins dataset showed a 1.60-fold increase (*p* < 0.001) in INHBB expression in prostatic intraepithelial neoplasia (PIN) compared to the healthy prostate (*n* = 25) (Figure 2C). 

Oncomine data for the expression of INHBC in the Liu Prostate dataset [34] showed elevated INHBC (fold change of 1.16; *p* = 0.003) expression in PCa when compared to the healthy prostate (Figure 2D). Oncomine analysis of the TCGA prostate dataset version 2012_10_12 also showed a significant increase (*p* < 0.0001) in INHBC expression compared to the prostate gland (Figure 2E); however, the expression change is minimal with a fold change of 1.02. 

Analysis of RNA-Seq data for INHBB using FireBrowse showed a 1.33-fold increase in INHBB expression in tumour samples compared to normal (Figure 2F). In contrast, the FireBrowse INHBC analysis showed a 0.388-fold decrease in INHBC expression in tumour samples compared to normal prostate samples (Figure 2G). 

### 3.3. Prostate Cell Lines Express Different Levels of INHBA, INHBB and INHBC

**Figure 3:**
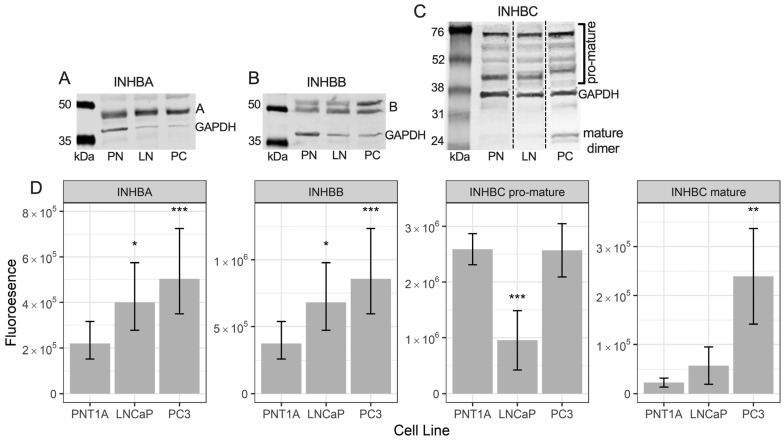
Representative Western blots of (**A**) INHBA, (**B**) INHBB, and (**C**) INHBC protein in PNT1A (PN), LNCaP (LN) and PC3 (PC) cells with GAPDH loading control. (**D**) Graphs showing relative fluorescent intensity of INHBA, INHBB, INHBC pro-mature multimers and INHBC mature dimer normalised to GAPDH in PNT1A, LNCaP and PC3 cells. Data is presented as the estimated marginal mean (emmean) ± 95% confidence limits. * *p* < 0.05, ** *p* < 0.01 and *** *p* < 0.001 relative to PNT1A cells from a General Linear Model analysis. Protein was isolated from one passage of each cell line for INHBA and INHBB and three separate passages for INHBC and quantified in three Western blots for each protein.

Western blot analysis for INHBA in the prostate cell lines showed a single band at approximately 45 kDa (Figure 3A). This antibody recognises the pro-region of the protein. LNCaP and PC3 cells had significantly higher expression of INHBA than PNT1A cells (Figure 3D).

The INHBB antibody used for Western blotting was made to a c-terminal (mature region) sequence. Western blotting for INHBB in protein extractions from the three prostate cell lines showed bands at approximately 50 and 55 kDa (Figure 3B) with weak bands at 12 kDa (not shown). Both LNCaP and PC3 cells exhibited significantly higher expression of INHBB when compared to PNT1A cells (Figure 3D).

The INHBC antibody recognises the mature region of the protein which was present in the Western blot as multimers of the pro- and mature regions of INHBC, between 45 and 75 kDa, and dimers of the mature region at approximately 25 kDa (Figure 3C). Bands of INHBC mature region monomer were visible in some blots at around 12 kDa but were very weak relative to the mature region dimer and higher molecular weight bands. PNT1A and PC3 cells expressed similar levels of INHBC pro-mature multimers and significantly more than the LNCaP cells while PC3 cells expressed more INHBC mature dimer than both PNT1A and LNCaP cells (Figure 3D).

### 3.4. Recombinant INHBC

Western blot analysis of our purified full-length recombinant INHBC, separated under reducing conditions, showed a band at approximately 12 kDa, the expected size of the mature chain monomer of INHBC (Supplementary Figure S2). A larger 55 kDa band was also present which is likely to consist of the unprocessed full-length protein, i.e., pro-region and mature region. The R&D Systems INHBC exhibited mature region monomer (12 kDa) and dimer (24 kDa) only and no pro-region as expected. 

### 3.5. Effect of Recombinant Activins on Prostate Cell Growth

**Figure 4:**
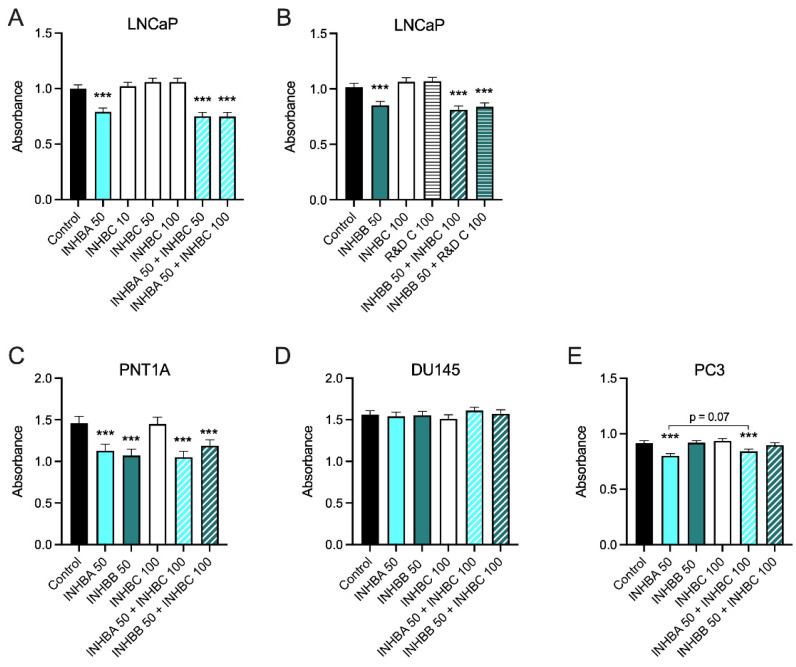
(**A**–**E**) MTS cell growth assays for prostate cell lines treated with exogenous recombinant INHBA, INHBB, INHBC, or INHBA or INHBB combined with INHBC (ng/mL). Data are presented as the emmean absorbance ± 95% confidence limits from a minimum of three separate biological replicates with three to six technical replicates per treatment per assay. Asterisks indicate significant differences between treated and untreated (control) cells *** *p* < 0.001 analysed by a NLM.

Recombinant INHBA inhibited growth of PNT1A, LNCaP and PC3 cells but not DU145 cells (Figure 4). INHBB inhibited growth of PNT1A and LNCaP cells but not PC3 or DU145 cells (Figure 4). In contrast, INHBC had no effect on growth of any of the cell lines either alone, or in combination with INHBA or INHBB, except for a trend towards an increase in absorbance when the combination of INHBA and INHBC was compared to INHBA alone in PC3 cells (*p* = 0.0697).

### 3.6. Overexpression of INHBB or INHBC Alters Growth and Migration of PCa Cell Lines

**Figure 5:**
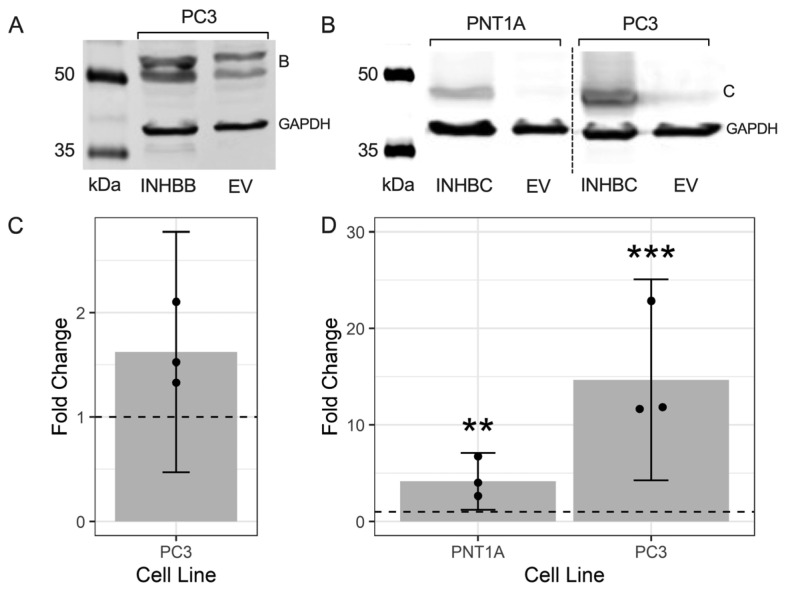
Representative Western blots showing the overexpression of (**A**) INHBB in transfected PC3 cells and (**B**) INHBC in transfected PNT1A and PC3 cell lines. Graphs show fold-change of the fluorescent intensity relative to the EV control cells (dashed line at 1.0) for (**C**) INHBB and (**D**) INHBC. Data presented as individual fold-change values (dots) and the emmean fold-change ± 95% confidence limits (grey bar and lines) from 3 Western blots. ** *p* < 0.01 and *** *p* < 0.001 analysed by a NLM.

**Figure 6:**
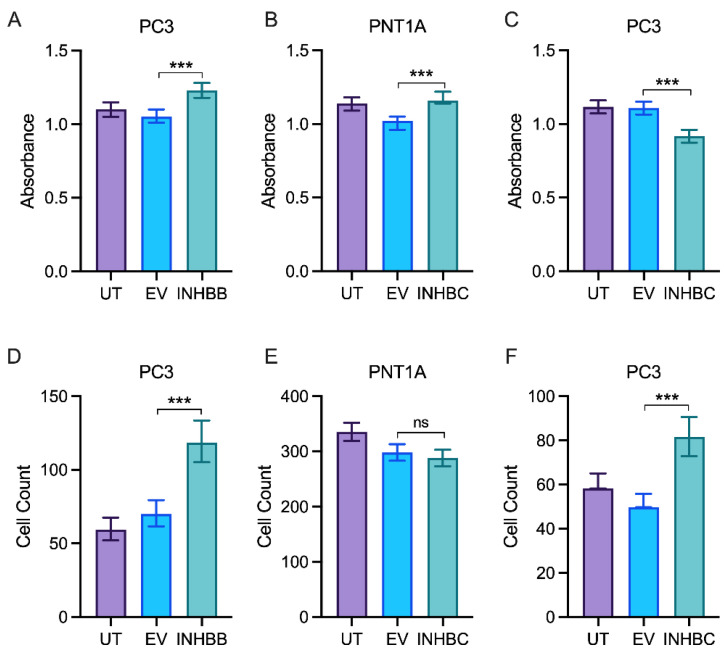
In vitro proliferation and migration assays for prostate cell lines either untransfected (UT), transfected with empty vector control (EV) or overexpressing INHBB or INHBC. MTS growth assay for (**A**) PC3 cells overexpressing INHBB, (**B**) PNT1A cells overexpressing INHBC and (**C**) PC3 cells overexpressing INHBC. Migration assays for (**D**) PC3 cells overexpressing INHBB, (**E**) PNT1A cells overexpressing INHBC and (**F**) PC3 cells overexpressing INHBC. Data are presented as estimated mean absorbance or cell count ± 95% confidence limits from a minimum of three separate passages with triplicate wells per assay. *** *p* < 0.001 analysed by NLM. ns: Insignificance is denoted by ns.

Western blot analysis showed expression of INHBB was higher in PC3 cell line engineered to stably overexpress INHBB when compared to the empty vector control cells (Figure 5A,C). However, the mean fold-change was not statistically different (*p* = 0.1882). INHBC was also overexpressed in PNT1A (*p* = 0.0018) and PC3 cells (*p* = 0.0001) when compared to empty vector controls (Figure 5B,D). PNT1A cells transfected with INHBB grew very slowly compared to those transfected with the negative control plasmid and were unable to be grown in sufficient numbers for further experiments, suggesting INHBB overexpression inhibits growth of or is genotoxic to these cells. The overexpression of INHBB in the PC3 cell line resulted in a robust increase in cell growth (*p* < 0.0001) and migration (*p* < 0.0001) when compared to the empty vector controls (Figure 6A,D). PNT1A cells overexpressing INHBC showed increased cell growth (*p* < 0.001) and no change in cell migration when compared to the empty vector controls (Figure 6B,E). In contrast, PC3 cells overexpressing INHBC showed decreased cell growth (*p* < 0.05) and increased cell migration (*p* < 0.0001) when compared to the empty vector controls (Figure 6C,F).

### 3.7. Response to Pathway Inhibitors Varies between Prostate Cell Lines

**Figure 7:**
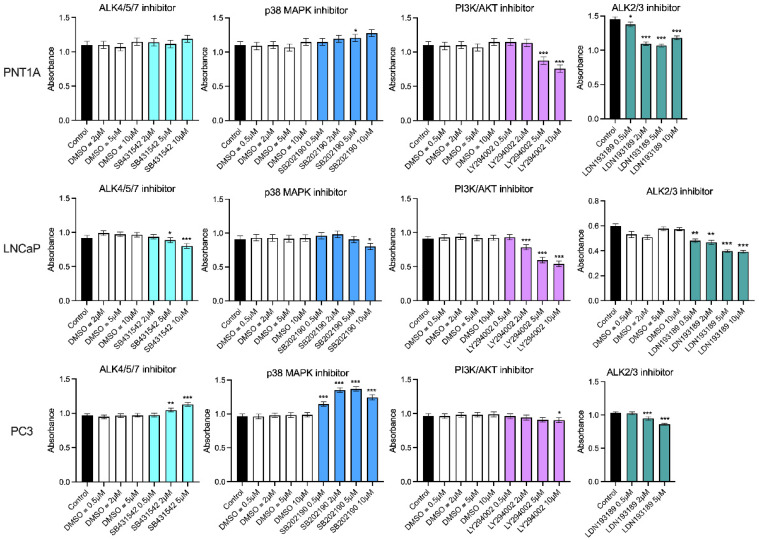
MTS growth assays for prostate cell lines treated with DMSO vehicle control or small molecule pathway inhibitors of ALK4/5/7 (SB431542), p38 MAPK (SB202190), PI3K/AKT (LY294002) and ALK2/3 (LDN193189). The DMSO was used at the equivalent dilution for each inhibitor dose. Data are presented as estimated mean absorbance ± 95% confidence limits from a minimum of three separate passages with three to six wells per assay. * *p* < 0.05; ** *p* < 0.01; *** *p* < 0.001 relative to the DMSO control, analysed by a NLM.

While the ALK4/5/7 (Smad2/3) pathway is reported to be the canonical signaling pathway for INHBA and INHBB, other pathways have also been shown to be activated by these ligands. Therefore, dose response growth assays were performed to determine the effect of the ALK4/5/7, p38 MAPK, PI3K/AKT and ALK2/3 pathway inhibitors on PNT1A, LNCaP and PC3 cell lines (Figure 7). Inhibition of ALK4/5/7 had no effect on PNT1A cell growth, but decreased LNCaP and increased PC3 cell growth at 5 μM and 10 μM. The p38 MAPK inhibitor increased growth in both PNT1A and PC3 cells with a greater effect on the PC3 cells. In contrast, inhibition of this pathway decreased growth of LNCaP cells. All three cell lines showed decreased growth in response to the PI3K/AKT inhibitor but the PC3 cells were less sensitive and only responded to the 10 μM dose (*p* < 0.05). The ALK2/3 pathway inhibitor was the only one that consistently decreased growth at the lowest concentrations across all three of the cell lines (Figure 7). There was no significant effect of the DMSO vehicle control at any of the concentrations tested in any cell line.

### 3.8. Summary of Results 

Table 3 presents a summary of the different expression levels and effects of activins on prostate cell lines and tissue.

## 4. Discussion

This study has demonstrated for the first time that INHBB and INHBC appear to have opposing roles in regulating PCa cell growth and migration. INHBB protein and RNA expression was increased in tumors with higher Gleason patterns or in PCa compared to normal prostate tissue. The PCa cell lines LNCaP and PC3 also had higher expressin of INHBB protein relative to immortalized prostate epithelial cells (PNT1A). In contrast INHBC protein expression decreased in higher-grade tumors and RNA expression appeared lower in tumor samples compared to normal prostate tissue in a relatively large data set of RNAseq data from the TCGA. 

In the absence other clinical data, such as patient survival, we analyzed the IRS of the TMA cores in relation to the highest Gleason pattern present. The morphological changes associated with increasing Gleason pattern represent cell and tissue level changes associated with more advanced cancer. While Gleason scores and the new ISUP grading system are good predictors of survival they still cannot accurately predict which tumors are faster growing or more invasive [35,36]. Tumor heterogeneity is high in PCa and this can be seen in the varied IRS scores across the different proteins and cases presented here. Therefore, any variation in IRS in individual cores relative to normal prostate tissue could indicate a protein is involved in tumor progression irrespective of the Gleason score assigned to the tumor biopsy, and these proteins warrant further study.

There were many cases with significantly increased IRS for INHBB in cores containing Gleason patterns 4 and 5 (Figure 1) and these tumors likely contain more cells undergoing rapid proliferation or epithelial-mesenchymal transition. Our current results indicate that INHBB could be used as a positive biomarker for distinguishing between latent and aggressive PCa tumors. Particularly if combined with decreasing INHBC expression as the IRS for these two proteins were negatively correlated and both changed when Gleason pattern 4 was present, compared to Gleason pattern 3 (Figure 1).

These results appear to differ to our previously published data that showed INHBC expression was higher, and INHBB expression was lower in tumors that had undergone extracapsular spread compared to normal tissue [16,37]. A digital scoring method was employed in the current study which removed the inter/intra-observer error associated with manual scoring methods used in earlier studies and an increased number of cases were assessed. In the previous studies, extracapsular spread was determined from the TNM grading for the patients which do not necessarily correspond to the Gleason scores of the individual biopsy cores on the TMA slide, due to heterogeneity within the tumor. Staining intensity generally differs between stromal and epithelial compartments and thus scores can vary depending on the composition of the core. Our current study has not differentiated between cell types within each biopsy core in order to identify biomarkers that could form the basis of a simple, robust test that does not require expert pathological examination to distinguish and score the different cell compartments. 

INHBA has been more extensively studied than the other activins and Kang et al. [38] reported increased INHBA protein and mRNA in primary PCa in patients who developed bone metastasis compared to those who did not develop bone metastasis. Chen et al. [15] have also shown expression of INHBA is elevated in PCa metastases and correlates with poorer survival. We did not observe any differences in INHBA expression, nor the activin receptors, ACVR2A (except for a slight decrease in IRS between patterns 3 and 4) and ACVR2B, between Gleason patterns (Figure 1) but PC3 cells expressed higher levels of INHBA than the immortalized epithelial PNT1A cells (Figure 3D). The mean IRS for inhibin-α (INHA) was higher in Gleason patterns 1 and 2 relative to normal tissue and then decreased again in higher Gleason patterns (Figure 1). INHA may be an early marker of PCa, although each pattern had some cases with high INHA IRS. In a study by Balanathan et al. [39], INHA staining intensity was higher in the stroma and benign regions of PCa tumors with extracapsular spread, but not the epithelial cancer regions, compared to organ confined cancer. There were no obvious changes to stromal INHA staining in our study, however, as mentioned previously, we did not score the different cell types separately. Similarly, BMP4 appears to have a transitional increase in expression in tumors exhibiting only Gleason patterns 1 or 2, followed by a decline in expression with patterns 4 and 5 (Figure 1). Whether the later stage tumors that continue to express higher levels of BMP4 are growing faster or slower is unknown but an increase in expression of this protein may be an early indicator of carcinoma. The remainder of the TGFB family proteins we have examined, SMAD2, SMAD3 and FST all decreased with increasing Gleason patterns compared to normal tissue but did not differ between patterns (Figure 1). Therefore, they would not be useful markers for differentiating between indolent and aggressive PCa. Shipitsin et al. [6] reported a negative correlation between SMAD2 expression and PCa lethality while Lu et al. [40] showed no correlation between SMAD2 and Gleason score but an increased expression of SMAD3 with increasing Gleason score. The loss of FST expression, an inhibitor of INHBA and INHBB may allow increased signaling by these proteins [41]. However, the concurrent loss of the SMAD signaling molecules that INHBA and INHBB have been shown to act through would negate this. Overall, our data highlight the important role of the TGFB family in maintaining normal prostate function as expression of many of these proteins was decreased in prostate tumors compared to normal prostate tissue.

Androgen receptor expression also declined with increasing Gleason grade (Figure 1). Published data for AR protein expression in PCa is contradictory and studies vary in their methodology. For example, some count nuclear staining or measure overall staining, others compare stromal vs. epithelial or primary cancer vs. metastasis. Several studies have, however, reported an inverse relationship between AR protein expression and Gleason grade, particularly when comparing stromal tissue to epithelial cells [42,43,44]. The current study did not differentiate between the stromal and epithelial compartments and the IRS for AR is significantly lower in all pathologies compared to normal prostate. Unfortunately, markers that decrease expression with increasing tumor grade are not as compatible with a robust prognostic test as those that increase expression. Expression of MKI67 and MYC increased in the higher Gleason pattern cores while BCL2 decreased (Figure 1). This is as expected as the first two are well known markers of proliferation associated with cancer growth while a decrease in BCL2 expression is associated with loss of apoptosis and increased proliferation. Mutations in *TP53* are commonly found in PCa but only some result in increased protein expression as observed in a small subgroup of biologically aggressive PCa [45]. The TP53 isoform ∆133TP53β has also been shown to be increased in higher grade PCa and is associated with immune cell infiltration [46]. These may explain why only a few of the TMA cases across different Gleason patterns in our study exhibited increased staining for TP53 (Figure 1). 

The findings from Oncomine and FireBrowse analysis of RNA expression are consistent with our protein expression results showing INHBB expression is higher, and INHBC is lower, in prostate tumours when compared to healthy prostate (Figure 2). This implies that increased expression of INHBB and decreased expression of INHBC may be associated with a poorer prognosis in PCa patients. 

Exogenous treatment with recombinant mature-region INHBA inhibited growth in all of the cell lines except for DU145 while both DU145 and PC3 cells were unaffected by treatment with exogenous mature-region INHBB (Figure 4). Addition of full-length recombinant INHBC had no effect on the growth of any of the prostate cell lines tested. The different responses of the cell lines may be due to differing endogenous expression levels of activins, their receptors or activin inhibitors such as FST [47,48]. McPherson et al. [47] showed INHBA inhibited LNCaP cells but in their study DU145 cells were also inhibited and PC3 cells did not respond to either INHBA or INHBB. In contrast, Simon et al. [49] have reported INHBA treatment of PC3 cells increased proliferation. However, this was in the presence of 1% FBS that had not been heat-inactivated. They also showed that INHBA antibody decreased non heat-inactivated serum induced proliferation of PC3 cells indicating this effect was due to an interaction between INHBA and another active component in serum. The proliferation assays in our current study were all performed in the presence of 2% heat inactivated FBS with the same media, conditions and endpoint which has enabled us to directly compare the responses of the different prostate cell lines to the different activins. 

The modification of PNT1A or PC3 cells to overexpress INHBC increased growth of PNT1A cells but decreased growth and increased migration of PC3 cells (Figure 6). This indicates that either our recombinant, purified INHBC protein is not bioactive or endogenous expression of INHBC is required for function. Overexpression of INHBB in PNT1A cells decreased the growth and survival of these cells which is in concordance with the effect of exogenous recombinant INHBB on these cells. In contrast, INHBB overexpression increased growth and migration of PC3 cells (Figure 6) suggesting these cells either respond to the endogenous presence of INHBB, or the full-length protein but not the mature region alone. The opposing effects of overexpression of INHBB and INHBC on the immortalized epithelial PNT1A cells and metastatic PCa PC3 cells indicates a switch in the function of these activins from inhibiting or regulating growth in normal epithelial cells to being pro-oncogenic in tumor cells. A similar switch in response to TGFB and INHBA in PCa has been previously described [50,51].

The in vitro assay data corresponds to the protein expression results for both the prostate biopsy cores and the cell lines which suggest an increase in INHBB expression in higher grade tumors and metastatic cell lines may be driving the development of more aggressive cancer, while a decrease in INHBC expression may be reducing growth inhibition in cases of prostate carcinoma. However, expression of INHBC pro- and mature-region was low in LNCaP cells but not PC3 cells, while PNT1A cells had low mature-region and high pro-region protein (Figure 3). 

The commercially available INHBC, consisting of the mature chain only, had no effect on LNCaP cell proliferation with or without INHBA or INHBB (Figure 4A,B). The pro-region of TGFB family proteins is known to be important for mediating folding and dimerization of the C-terminal mature domains, binding to extracellular matrix and regulation of signaling [41,52,53]. We hypothesized that the activity of INHBC may require the presence of the N-terminal pro-region, as is the case for cumulin, another TGFB family member [24]. However, our purified, full length, recombinant INHBC containing both the pro-region and mature region proteins was unable to inhibit INHBA or INHBB suppression of growth in LNCaP cells but did trend towards an inhibition of INHBA function in PC3 cells. This differs to results previously reported in LNCaP cells using an unpurified INHBC preparation produced in CHO cells which was able to block INHBA inhibition of LNCaP cell growth [13,14]. CHO cells secrete many factors that can alter cell growth and therefore the effect reported from this INHBC preparation could potentially have been due to the presence of contaminating proteins. Goebel et al. [54] have also demonstrated that mature-region INHBC does not directly antagonize INHBA signaling. However, inhibin-α knock-out mice that have increased INHBA and develop testicular and ovarian tumors, were shown to have reduced tumor formation when modified to overexpress INHBC [17]. Other studies have reported that INHBC can reduce the formation of homodimeric INHBA and increase heterodimeric INHBAC which has a lower affinity for the ACVR2B receptor [14,55]. Marino et al. [22] have also shown that INHBC can directly bind the ACVR2A and 2B receptors but does not alter SMAD2/3 or ERK signaling, indicating INHBC may block INHBA receptor binding.

Inhibition of the ALK4/5/7 signaling pathway had no effect on PNT1A cell growth indicating this pathway is not very active in normal prostate epithelial cells (Figure 7). This may be because INHBA that is known to activate this pathway had low expression in PNT1A cells. Blocking the ALK4/5/7 pathway in PC3 cells however, increased growth, likely due to inhibition of INHBA signaling which would normally decrease growth of these cells. Unexpectedly, LNCaP cells had decreased growth when exposed to the ALK4/5/7 inhibitor. MAPK inhibition decreased growth of both PNT1A and PC3 cells but again had the opposite effect on LNCaP cells. The ALK2/3 inhibitor was the only one to significantly inhibit growth of all three prostate cell lines and therefore the use of the LDN193189 small molecule inhibitor for the treatment of PCa should be further explored. 

## 5. Conclusions

To summarize, our results suggest that combining quantification of INHBB and INHBC in prostate biopsies may enable the identification of tumors containing cells that will be particularly malignant. These, and other TGFB family proteins, could provide more accurate predictions of clinical outcome and guide decision making in relation to surgery, active surveillance, or watchful waiting, and thus improve life expectancy and quality for PCa patients. Inhibition of INHBB expression and/or targeting the ALK2/3 pathway may also be novel therapeutic targets for the treatment of PCa. Further work is needed to elucidate the mechanisms of INHBC cell signaling and to corroborate the TGFB family expression differences observed in a larger cohort of PCa patients with more comprehensive clinical data.

## Figures and Tables

**Table 1:** Antibodies used for immunohistochemistry.

Antibody	Supplier	Cat. No	µg/mL or Dilution	Species & Clonality
INHBA	Abcam	ab56057	20	Rabbit polyclonal
INHBB	R & D Systems	MAB659	2.5	Mouse monoclonal
INHBC	Abcam	ab73904	1	Mouse monoclonal
INHA	Abcam	ab81234	0.2	Rabbit polyclonal
ACVR2A	Abcam	ab10595	5	Goat polyclonal
ACVR2B	Abcam	ab76940	12.5	Mouse monoclonal
SMAD2	Abcam	ab47083	5	Rabbit polyclonal
SMAD3	Abcam	ab40854	14	Rabbit monoclonal
FST	Abcam	ab157471	8	Rabbit monoclonal
BMP4	Abcam	ab39973	1	Rabbit polyclonal
AR	Abcam	ab3509	1:300	Rabbit polyclonal
MKI67	Abcam	ab66155	2.5	Rabbit polyclonal
MYC	Abcam	ab32	2.5	Mouse monoclonal
TP53	Abcam	ab26	5	Mouse monoclonal
BCL2	Abcam	ab7973	0.6	Rabbit polyclonal
Negative mouse	DAKO	X0931	12.5	Mouse monoclonal
Normal goat	Santa Cruz	Sc-2028	5	Goat IgG

Abcam (Cambridge, UK); R & D Systems (Minneapolis, MN, USA); DAKO (Santa Clara, CA, USA); Santa Cruz (Dallas, TX, USA).

**Table 2:** Antibodies used for Western blots.

Antibody	Supplier	Catalogue No	Dilution	Species & Clonality
INHBA (pro-region)	Abcam	ab128958	1:1000	Rabbit Monoclonal IgG1
INHBB (mature)	Abcam	ab128944	1:2000	Rabbit polyclonal IgG
INHBC (mature)	Abcam	ab73904	1:1000	Mouse monoclonal IgG1
GAPDH	Abcam	ab9484	1:6000	Mouse monoclonal IgG2b
GAPDH	Abcam	ab181602	1:6000	Rabbit monoclonal
Anti-rabbit	LI-COR	C20322-01	1:10,000	800 CW
Anti-rabbit	LI-COR	C10628-01	1:25,000	680 LT
Anti-mouse	LI-COR	926-32350	1:10,000	IgG1 800 CW
Anti-mouse	LI-COR	926-68052	1:25,000	IgG2b 680 LT

**Table 3:** Summary of INHBA, INHBB, INHBC expression and function in prostate cells.

Prostate Tissue or Cell Line	Activin Expression			Exogenous Treatment			Overexpression			
				Growth			Growth		Migration	
	A	B	C-Mat	A	B	C	B	C	B	C
Normal	mod	mod	mod							
Gleason 4 & 5	mod	↑	↓							
PNT1A	low	low	low	↓	↓	=	↓	↑	-	=
LNCaP	↑	↑	low	↓	↓	=	-	-	-	-
PC3	↑	↑↑	↑	↓	=	=	↑	↓	↑	↑

A (INHBA); B (INHBB); C-mat (INHBC mature region); C (INHBC); ↑ (increased); ↓ (decreased); mod (moderate); = (no change); - (not done).

## Data Availability

The data presented in this study are available on request from the corresponding author.

## Supplementary Materials

The following supporting information can be downloaded at: https://www.mdpi.com/article/10.3390/cancers15010147/s1, Figure S1: Representative images of immunohistochemical staining for each TGF-β family protein in normal prostate tissue (left) and ISUP Gleason grade 5 prostate cancer tissue cores (right). INHBA, INHBB, INHBC, inhibin-α (INHA), activin receptor type 2A (ACVR2A), activin receptor type 2B (ACVR2B), mothers against decapentaplegic homolog 2 or 3 (SMAD2, SMAD3), follistatin (FST), bone morphogenetic protein 4 (BMP4), androgen receptor (AR), marker of proliferation Ki-67 (MKI67), MYC proto-oncogene (MYC), tumor protein p53 (TP53), B-cell CLL/lymphoma 2 (BCL2); Figure S2: Western blot of recombinant INHBC from R&D Systems and our laboratory (rActC) showing presence of pro-mature and mature-region bands in our full-length recombinant protein.
