# Supplementary material for: Activin B and Activin C Have Opposing Effects on Prostate Cancer Progression and Cell Growth
Source: Cancers (Basel). 2022 Dec 27;15(1):147. doi: 10.3390/cancers15010147 (PMC9817897; doi:10.3390/cancers15010147)
Supplement: Supplementary file 1 [file cancers-15-00147-s001.zip › cancers-2037030-supplementary.pdf]

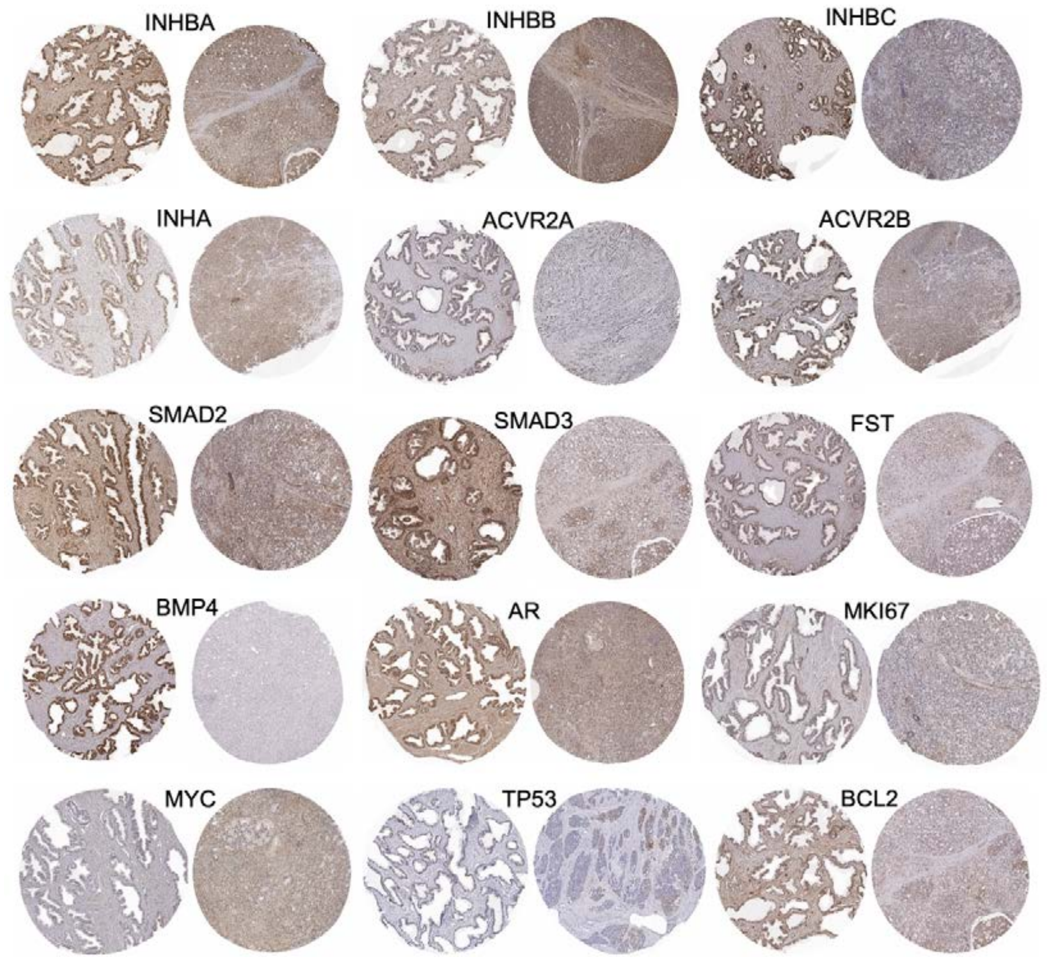

Supplementary Figure 1. Representative images of immunohistochemical staining for each TGF-β family protein in normal prostate tissue (left) and ISUP Gleason grade 5 prostate cancer tissue cores (right). INHBA, INHBB, INHBC, inhibin-α (INHA), activin receptor type 2A (ACVR2A), activin receptor type 2B (ACVR2B), mothers against decapentaplegic homolog 2 or 3 (SMAD2, SMAD3), follistatin (FST), bone morphogenetic protein 4 (BMP4), androgen receptor (AR), marker of proliferation Ki-67 (MKI67), MYC proto-oncogene (MYC), tumor protein p53 (TP53), B-cell CLL/lymphoma 2 (BCL2).

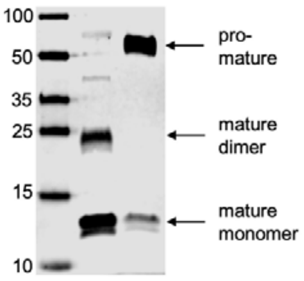

Supplementary Figure 2. Western blot of recombinant INHBC from R&D Systems and our laboratory (rActC) showing presence of pro-mature and mature-region bands in our full-length recombinant protein.
